# Factor structure and psychometric properties of the Jordanian version of the depression anxiety stress scale (DASS‐21)

**DOI:** 10.1002/npr2.12442

**Published:** 2024-04-08

**Authors:** Khaled A. Al‐Dassean, Odeh S. Murad

**Affiliations:** ^1^ Al‐Balqa Applied University, Al‐Shoubak University College Al Shoubak Jordan

**Keywords:** depression anxiety stress Scale‐21, factor structure, Jordanian version

## Abstract

**Aim:**

Early diagnosis of health conditions such as depression, anxiety, and stress, which have been recognized as global health issues, is essential for providing psychological support to people who experience negative emotions. Therefore, it is important to identify a reliable method for diagnosing depression, anxiety, and stress. To this end, this study investigated the factor structure and evaluated the psychometric properties of the Depression Anxiety Stress Scale‐21 (DASS‐21) in Jordan.

**Methods:**

A university sample of 336 Jordanians completed the Arabic version of the DASS‐21 and several psychopathology measures. Cross‐sectional study and confirmatory factor analysis (CFA) were applied to achieve the study's aims.

**Results:**

CFA favored the bifactor model compared to the other four models. In contrast, the Beck Anxiety Inventory (BAI) and the Beck Depression Inventory‐II (BDI‐II) strongly correlated with the DASS‐21, indicating that the DASS‐21 has adequate convergent and divergent validity. Subsequently, the scale's discriminant validity was tested using the heterotrait–monotrait (HTMT) correlation ratio, which was found to be relatively insufficient. While DASS‐21 showed strong reliability, Cronbach's α and McDonald's omega values ranged between 0.83 and 0.93.

**Conclusion:**

The bifactor model best fits the Jordanian sample data. The DASS‐21 has optimum psychometric properties; therefore, it can be used to assess the general distress experienced by the Jordanian community during research investigations and in non‐clinical settings.

## Abstract 2

The current study investigated the factor structure and evaluated the psychometric properties of the Depression Anxiety Stress Scale‐21 (DASS‐21) in Jordan. A university sample of 336 Jordanians completed the Arabic version of the DASS‐21 and several psychopathology measures. The results revealed that the bifactor model best fits the Jordanian sample data, and the DASS‐21 has adequate convergent and divergent validity.

## Graphical abstract

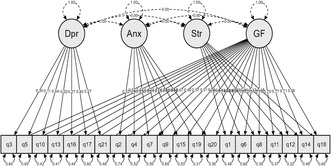

## 1 INTRODUCTION

Depression and anxiety are comorbid disorders with similar and distinguishing characteristics. Their prevalence and the inadequacy of traditional self‐report methodologies to distinguish between them have been well documented.^1^ Depression and anxiety are prevalent, non‐psychotic mental disorders that affect people of all ages across all countries.^2^, ^3^ Therefore, early detection of these issues in the healthcare system is critical for identifying individuals who may benefit from focused therapy, improving their prognosis, and lowering community disability. According to the recent estimates provided by the World Health Organization, over 615 million individuals worldwide struggle with depressive and anxiety disorders, costing the global economy approximately one trillion dollars each year.^4^ Many scales are available to assess psychological disorders such as depression and anxiety, for example, the Beck Anxiety Inventory (BAI) and the Beck Depression Inventory (BDI). However, only one tool is currently available to simultaneously measure depression, anxiety, and stress—the Depression Anxiety Stress Scale (DASS).^5^ Therefore, it was important to implement the DASS in order to eliminate measurement overlap between depression and anxiety, which jeopardizes the purity of independent depression and anxiety measures.^6^

The Depression Anxiety Stress Scale (DASS‐21) is a short version of the DASS, which comprises 21 items. It is an easy‐to‐use scale that can be used in research and clinical settings. The psychometric characteristics of the DASS‐21 have been tested in a variety of populations with generally positive results, and studies have found that the different models of the DASS‐21 have strong internal consistency.^7^, ^8^, ^9^, ^10^, ^11^, ^12^ Previous studies (for example,^13^, ^14^, ^15^, ^16^) revealed that the DASS‐21 scale has optimum psychometric properties in the various cultural settings wherein they are applied, regardless of whether they are applied on normal people or clinical patients.

Furthermore, studies have revealed good reliability coefficients using various methods, such as Cronbach's α and test‐retest.^1^, ^2^, ^3^, ^17^ The scale's validity was confirmed in various ways, such as convergent, divergent, and discriminant validity, by examining the scale's ability to distinguish between normal and psychologically disturbed people.^18^, ^19^ The construct validity was also tested in many studies, thereby confirming its association with well‐known scales, such as the Beck Depression Inventory (BDI), the Beck Anxiety Inventory (BAI), and other related scales.^1^, ^20^ The results of previous studies vary in terms of the factor structure. Some studies that used exploratory factor analysis (EFA) revealed the presence of three qualitative factors, representing the three subscales of the original Australian scale (depression, anxiety, and stress).‐^5^, ^21^, ^22^ On the other hand, some other studies revealed a factor for general distress in addition to the three specific factors.^23^

In contrast, the studies conducted by Tran et al. and González‐Rivera et al.^2^, ^24^ revealed one general factor for negative emotions with which all items of the scale are saturated. Studies that used confirmatory factor analysis (CFA) also differed in terms of the proposed models that represent possible scale structures, as many of them confirmed the original Australian model with three correlated factors.^3^, ^9^, ^12^, ^13^, ^14^, ^15^, ^21^, ^25^, ^26^, ^27^, ^28^, ^29^, ^30^

Some studies have supported the tripartite factor structure.^17^, ^31^ On the other hand, some previous studies supported the bifactor model.^1^, ^10^, ^23^, ^32^, ^33^, ^34^ Most previous studies conducted on DASS‐21 were focused on the Western culture, and only a few studies were conducted with reference to the Eastern Arab culture.^34^, ^35^, ^36^, ^37^, ^38^ The studies used various methods to validate the scale. Consequently, their results were mixed, and some studies did not assess the factor structure (for example,^38^). In the study conducted by Alansari in Kuwait,^39^ the EFA and CFA results revealed three correlated factors, as highlighted in the original version as well.

On the other hand, the studies conducted by Abdelati^37^ and Ali and Green^40^ in Egypt revealed the presence of two factors and one factor, respectively, of DASS‐21. Al‐Zahrani^36^ evaluated a Saudi version of the DASS‐21 with a focus on nursing staff and revealed the presence of three correlated factors. Subsequently, after applying modification procedures to the model, it led to the deletion of item 20. Furthermore, in Algeria, Rasheed^35^ sought to verify the properties of the factor structure of the DASS‐21 scale among secondary school students. The results of the CFA revealed three second‐order factor models. However, the study included unjustified paradoxes when modifying the model, such as allowing measurement errors to be correlated with items that did not belong to the same subscale. In addition, a recent study performed by Zanon et al.,^34^ involving a sample from the United Arab Emirates, found that the bifactor model was the best fit for the DASS‐21 structure. Notably, none of the Arab studies investigated the discriminant validity of the DASS‐21 subscales. To the researcher's knowledge, no study has investigated the psychometric properties and factor structure of the DASS‐21 in Jordan. Although there are several scales available in Jordan to assess depression, anxiety, and stress, there is currently no scale that combines these disorders in a concise, easy‐to‐apply style suited to use in research studies or clinical diagnosis.

### 1.1 Present study

The current study focused on validating the factor structure of the DASS‐21 using CFA by comparing several factor models (one general factor, two‐factor, three correlated factors, hierarchal and bifactor models). Furthermore, it aimed to evaluate the psychometric properties of the DASS‐21 scale in a non‐clinical sample of the Jordanian population. The present study also assessed convergent and divergent validity through correlation with the BDI‐II and the BAI. Moreover, the discriminant validity of the subscales was evaluated using the heterotrait–monotrait (HTMT) criterion, a relatively new statistical method commonly used in studies^41^ that has been shown to provide better sensitivity and specificity than most other statistical methods, such as cross‐loading assessment and Fornell‐Lacker method. This study also verified the reliability indicators for the scale as a whole and their three subscales using Cronbach's α and McDonald's omega.

## 2 METHODS

### 2.1 Participants and procedure

In total, 336 students were recruited from Al‐Balqa Applied University in Jordan at the following two academic levels: a technical diploma of 44.4% and a bachelor's of 55.6%. Subsequently, it was found that five questionnaires were unsuitable for statistical analysis due to the many unanswered questions. Therefore, the final number of students was 331, with an average age of 21.9 years, standard deviations of 3.3 years, and a female participation percentage of 63.7%. Data was collected in two ways—first, by paper and pencil, and second, electronically. Overall, using the first method, 190 questionnaires were filled out inside the classrooms at Al‐Shoubak University College under the supervision of the first author during the 2022–2023 summer semester. On average, the participants took 15–20 min to complete the questionnaires. The remaining 141 participants were recruited electronically through advertisements in student groups and with the assistance of the heads of the academic departments at the university.

A web page was created using Google Forms, inviting students to participate in the study. Multiple responses were prevented, thereby limiting the possibility of selection bias. Moreover, prior consent was secured from the students for their participation. The students were informed about the research objectives and that they could withdraw from the study at any point in time they wished, as participation was voluntary and did not entail rewards or prizes. The anonymity of the participants and the confidentiality of the data were preserved. Jordanian adults above 18 with a high school diploma or higher educational qualification were eligible to participate in this study. International students of different nationalities were excluded. Permission to use the scale was secured by email communication from Professor Peter Lovibond before the commencement of the study. The study was conducted in adherence to the Helsinki Treaty's ethical standards and those established by the Protocol of the Practical Research Ethics Committee in Jordanian higher education institutions.

## 3 MEASURES

### 3.1 The depression anxiety stress Scale‐21 (DASS‐21)

A validated Arabic translation Moussa et al.^42^ of the original DASS‐21 scale Lovibond and Lovibond^5^ was obtained from the DASS official website. A self‐report questionnaire with 21 items divided into three subscales was used to assess depressive symptoms such as low self‐esteem, life devaluation, lethargy, hopelessness, and self‐deprecation. The most common symptom of anxiety is physiological arousal, whereas stress symptoms include difficulty experienced in relaxing, general stress, impatience, irritation, and insomnia.^43^ During the last week's session, participants' degree of approval for the symptoms was marked on a 4‐point Likert scale ranging from 0 (not applicable to me at all) to 3 (applicable to me often or most of the time). Higher scores reflected higher levels of symptom support and more psychological distress in the adult population. In addition, the initial evaluation of the translated version revealed adequate validity and reliability indicators.

### 3.2 The Beck depression Inventory‐II (BDI‐II)

Al‐Da'asin^44^ adapted the BDI‐II to the Arabic version used in this study. It is a 21‐item scale that aids in assessing the emotional, cognitive, motivational, vegetative, and psychomotor components associated with depression. The participants were asked to choose the sentence that best described their feelings and conditions in the previous 2 weeks from a graduated sequence of four possibilities expressing the intensity of depression, ranging from 0 to 3, with sadness gradually rising in intensity. The Arabic version of the BDI‐II exhibited high psychometric properties and good internal consistency coefficients. Cronbach's α for the scale was 0.86 in the current investigation.

### 3.3 The Beck anxiety inventory (BAI)

BAI is a 21‐item self‐report questionnaire that assesses anxiety levels.^45^ Four options representing anxious symptoms were presented in ascending order of severity from 0 to 3 for each item, with a high anxiety level indicated by the highest score. The participants were asked to choose the term that best described how they felt about their symptoms throughout the previous week. The Arabic version of the scale has good psychometric properties.^46^ Cronbach's *α* was 0.94 in the current study.

### 3.4 Statistical analysis

The IBM SPSS V‐23 and JASP programs were used to analyze the data. Several descriptive statistics were calculated to describe the characteristics of the scale items. Skewness and kurtosis values were used to examine the normality of the scores for each item. According to Kim,^47^ the non‐normality of the large sample data can be indicated by skewness values larger than two and kurtosis values larger than seven. Pearson correlation coefficient was calculated to estimate the convergent and divergent validity between the DASS‐21 and related measures (BDI‐II and BAI).

The discriminant validity was also validated using the HTMT approach proposed by Henseler et al.^41^ through a Monte Carlo simulation study. Consequently, it was found that this method has the highest percentage of specificity and sensitivity compared to other methods, such as the cross‐loading assessment and the Fornell‐Lacker method. As an HTMT value close to 1 indicates a lack of discriminative validity, the HTMT value was compared to a pre‐established cutoff point of 0.85 or 0.90. The criterion of 0.85 was adopted in the current study, as it is more conservative and provides the highest level of sensitivity.^41^ If the value exceeds 0.85, it indicates a lack of discriminant validity. It is noteworthy that the current study used the terms divergent and discriminant validity differently concerning the procedures for assessing them, where divergent validity refers to the extent to which the DASS‐21 scales differ from external tests (BAI, BDI‐II) as measured by the Pearson correlation coefficient, and discriminant validity refers to the extent to which the ability of the DASS‐21 subscales to distinguish between depression, anxiety, and stress using the HTMT approach.

CFA was used along with the maximum likelihood method to verify the factor structure of the DASS‐21 instrument. Five models were evaluated using CFA in line with previous findings and following the recommendations of Reise et al.^48^ These models were as follows: (1) original model consisting of three related factors (depression, anxiety, and stress) proposed by Lovibond and Lovibond^5^; (2) a bifactor model in which each of the 21 items is restricted to loading on a general factor and one of the three specific factors (depression, anxiety and stress); (3) the hierarchical model proposed by Willemsen et al.^31^ wherein each of the 21 items is constrained to load on a general factor and two specific factors for the depression and anxiety items; (4) two‐factor model (wherein the depression items were loaded on one factor, whereas anxiety and stress were loaded on another factor); and (5) one‐factor model (with all items loading on a general factor). The following goodness‐of‐fit indices were used to compare the models: Chi‐square, standardized root mean square residual (SRMR), root mean square error of approximation (RMSEA), Comparative Fit Index (CFI), Non‐Normed Fit Index (NNFI), and Akaike information criterion (AIC). The following criteria were used to judge the values of the previous indicators: Chi‐square = not significant, even though it is affected by sample size; CFI and NNFI >0.90; RMSEA <0.06; SRMR <0.08 Adequate; and <0.05 good fit.^49^ The AIC index and ΔCFI were used to compare different models, as the models with the lowest AIC values were the ones that had the best goodness‐of‐fit. Higher values indicated less parsimony in the models, and ΔCFI >0.01 indicated differences between models.^49^, ^50^ Although a few sample size guidelines were required for CFA, the participation of 265 students was considered adequate for this investigation to achieve a power of 0.80.^51^

The Cronbach's α and McDonald's omega coefficients were calculated for the DASS‐21 to verify reliability. In this context, a value of 0.8 or more indicated good internal consistency.^52^ Moreover, the hierarchical omega ω_H_ procedure was used to assess the reliability of the bifactor model. This method allowed us to assess the reliability of the DASS‐21 subscales while controlling the effect of the general factor.^53^

## 4 RESULTS

### 4.1 Descriptive statistics and item characteristics of DASS‐21

Table 1 shows item‐level and scale descriptive statistics (mean, standard deviation, skewness, and kurtosis). The mean item scores ranged between 0.72 (SD = 0.97) and 1.84 (SD = 1.05), whereas skewness (ranging from −0.42 to 1.15) and kurtosis (ranging from −1.55 to 0.16) did not exceed Kim's recommended cutoffs for normal distribution.^47^ Many depression scale items were slightly negatively skewed, whereas the stress scale was completely negatively skewed. The corrected item total correlations in the three subscales ranged from 0.45 (item 9) to 0.71 (item 16), whereas the mean inter‐item correlations were consistently well above 0.40, which is regarded as an adequate value for narrow constructs.^54^

**TABLE 1:** Descriptive statistics of DASS‐21 scales & items.

Scale		Mean	SD	Kurtosis	Skewness	CITC
Depression	3	1.31	1.04	−1.11	0.24	0.55
	5	1.23	1.03	−1.04	0.33	0.47
	10	1.12	1.12	−1.14	0.47	0.67
	13	1.75	1.03	−1.09	−0.29	0.62
	16	1.65	1.12	−1.32	−0.20	0.71
	17	0.72	0.97	0.16	1.15	0.52
	21	1.51	1.21	−1.55	−0.03	0.67
	Total	9.33	5.40	−0.79	0.16	MIIC = 0.43
Anxiety	2	1.26	1.09	−1.24	0.29	0.47
	4	1.27	1.14	−1.33	0.31	0.61
	7	1.39	1.14	−1.41	0.17	0.57
	9	1.50	1.14	−1.41	0.00	0.45
	15	1.21	1.12	−1.29	0.34	0.62
	19	1.37	1.16	−1.43	0.17	0.69
	20	1.26	1.13	−1.28	0.33	0.67
	Total	9.27	5.62	−0.79	0.16	MIIC = 0.42
Stress	1	1.69	1.02	−1.01	−0.21	0.59
	6	1.54	1.11	−1.33	−0.07	0.54
	8	1.84	1.05	−1.07	−0.42	0.68
	11	1.62	1.03	−1.15	−0.09	0.70
	12	1.56	1.03	−1.16	−0.03	0.67
	14	1.49	1.00	−1.07	0.06	0.63
	18	1.51	1.15	−1.44	0.00	0.51
	Total	11.27	5.41	−0.68	−0.22	MIIC = 0.46
DASS‐21 total		29.88	14.91	−0.59	−0.023	MIIC = 0.40

Abbreviations: CITC, corrected item total correlation; MIIC, mean inter‐item correlation; SD, standard deviation.

### 4.2 CFA of competing models

CFA tested competing models to reveal the nature of the factor structure of the DASS‐21; these results are presented in Table 2. The three‐factor structure model proposed by Lovibond and Lovibond^5^ revealed a poor fit to the Jordanian data. The correlation between three factors in this model was very strong: depression anxiety = 0.77; depression stress = 0.95; and anxiety stress = 0.85. The one and two‐factor models had the lowest fit indices and did not match the acceptable fit. The fit indices for the alternative hierarchical model were proposed by Willemsen et al.,^31^ resembling the tripartite model, which had an adequate fit, RMSEA <0.08, CFI = 0.92. However, the bifactor model had a good fit. When comparing these two models, we found that the bifactor model had the highest fit χ2 = 361.62 (df = 168, *p* < 0.01), CFI = 0.94, and RMSEA = 0.059. The ΔCFI between the bifactor and tripartite models was 0.018, indicating that the bifactor model fits the observed data best. Furthermore, the AIC indicates the model's parsimony with the lowest value. The omega hierarchy for the overall score of the bifactor model was 0.89, thereby indicating the presence of a general factor.

**TABLE 2:** Confirmatory factor analysis.

Model	*X* ^2^	df	*p*	CFI	NNFI	RMSEA	SRMR	AIC
1: Three Factors	509.34	186	0.001	0.900	0.887	0.072	0.051	18054.01
2: Bifactor model	**361.62**	**168**	**0.001**	**0.940**	**0.925**	**0.059**	**0.039**	**17942.62**
3: Tripartite	424.57	172	0.001	0.922	0.904	0.067	0.042	17997.57
4: Two Factors	638.28	188	0.001	0.853	0.836	0.085	0.056	18686.90
5: One Factor	648.00	189	0.001	0.858	0.842	0.086	0.055	18426.54
Cutoff score				>0.90	>0.90	<0.06	<0.08	

*Note*: The best‐fitting model is highlighted in bold. There are values with fit statistics and no significance values.

Abbreviations: AIC, Akaike's Information Criterion; CFI, Comparative Fit Index; df, degrees of freedom; NNFI, Non‐Normed Fit Index; RMSEA, root mean square error of approximation; SRMR, standardized root mean square residual.

**FIGURE 1:**
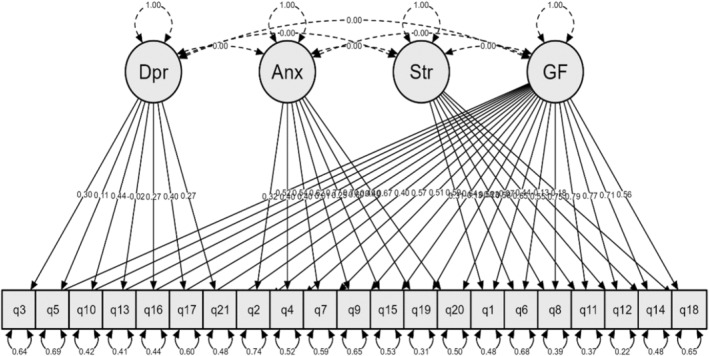
Depicts a conceptual visualization of the bifactor factor model evaluated in the CFA.

Therefore, 89% of the variances of all items may be assigned to the general factor. Figure 1 depicts the conceptual visualization of this model. An in‐depth investigation of the factor loadings reveals that the loadings of the items on the general factor were high, ranging from 0.44 (Item 2) to 0.81 (Item 11). This indicates that the general factor accounts for 20 to 67% of each item's variance. While the loadings of the items on the specific factors ranged from 0.007 (Item 9) to 0.69 (Item 19), this indicates that only 0.00005% to 48% of the variance of each item can be attributed to the specific factors. An examination of the loadings in Table 3 indicates that, in the bifactor model, the subscale loadings are generally lower after controlling for the general factor. The loadings on the depression factor were significant but not high, except for items 5 and 13, which were not significant. In addition, the loadings for the anxiety item were significant for six items, except for item 9. However, the stress factor only includes the two least significant items, item 1 and item 12. Notably, there are four items with negative loadings on the stress factor.

**TABLE 3:** Bifactor solution of the Jordanian DASS‐21 scale.

Item	General factor	Specific factors			
		Depression	Anxiety	Stress	Residual variance
3	0.540	0.314			0.688
5	0.556	*0.117*			0.729
10	0.677	0.485			0.508
13	0.786	*−0.061*			0.432
16	0.779	0.304			0.548
17	0.478	0.386			0.566
21	0.807	0.330			0.693
2	0.439		0.349		0.889
4	0.649		0.453		0.677
7	0.578		0.451		0.764
9	0.676		*0.007*		0.844
15	0.716		0.283		0.660
19	0.669		0.694		0.415
20	0.656		0.453		0.633
1	0.664			0.315	0.501
6	0.608			*−0.143*	0.836
8	0.788			−0.244	0.429
11	0.815			*0.071*	0.392
12	0.794			0.453	0.233
14	0.710			*−0.133*	0.484
18	0.650			−0.207	0.866
*ω*_H_	0.89	0.13	0.23	0.004	

*Note*: All factor loading are significant at 0.05 with exception of those in italics, which have *p* >0.05.

Abbreviation: *ω*_H_, Omega Hierarchical.

These findings are supported by the omega hierarchical statistical analysis, which clearly shows that the high percentage of the variance in total items is due to the general factor. This is because, if we control for the effect of this factor, the reliability of the specific factors estimated by the omega hierarchy is very low (0.004–0.23) (Table 3).

### 4.3 Reliability of the Jordanian version of DASS‐21 and related measures

The internal consistency reliability results of the three subscales indicate that each scale has strong internal consistency reliability. Cronbach's α coefficients ranged from 0.83 to 0.85, and McDonald's omega ranged from 0.84 to 0.85, with a total score (*α* and *ω*) of 0.93 (Table 4). Strong correlations, varying from 0.67 to 0.79, *p* = 0.01 (mean *r* = 0.73, that is, 53% of common variance), were found between the three DASS‐21 subscales, as the correlations between each DASS‐21 subscale and the overall score were also examined (ranging from 0.89 to 0.93). Table 5 shows these results.

**TABLE 4:** Reliability of the Jordanian version of the DASS‐21 (Alpha & Omega) and related measures.

Scale	*α*	95% CI	*ω*	95% CI
DASS‐21	0.93	0.92–0.94	0.93	0.92–0.94
1. Depression	0.84	0.82–0.87	0.85	0.83–0.87
2. Anxiety	0.83	0.81–0.86	0.84	0.81–0.86
3. Stress	0.85	0.83–0.88	0.85	0.83–0.88
BDI‐II	0.91	0.90–0.93	0.91	0.90–0.93
BAI	0.94	0.93–0.95	0.94	0.93–0.95

Abbreviations: *α*, Cronbach's alpha; *ω*, McDonald's Omega; 95% CI, 95% confidence interval.

**TABLE 5:** Correlations between the DASS‐21, (BDI‐II) and (BAI), as well as correlations between DASS‐21 subscales and total score.

	BDI‐II	BAI	1	2	3	DASS‐21 total
1‐DASS‐21 Depression	0.71**	0.63**	1	0.67**	0.79**	0.90**
2‐DASS‐21 Anxiety	0.57**	0.73**		1	0.74**	0.89**
3‐DASS‐21 Stress	0.65**	0.70**			1	0.93**
DASS‐21 total	0.71**	0.76**				1

Abbreviations: BAI, Beck Anxiety Inventory; BDI‐II, Beck Depression Inventory–Second Edition.

** *p* < 0.01.

### 4.4 Convergent and divergent validity

The DASS‐21 scales (depression, anxiety, and stress) were highly correlated in the expected direction with the BDI‐II and BAI inventories (Table 5). The DASS‐21 anxiety subscale and the BAI had the highest unique correlation (*r* = 0.73) when comparing the correlation with BDI‐II (*r* = 0.57, *z* = 3.4, *p* < 0.0001). The DASS‐21 depression subscale and the BDI‐II had the second‐highest remarkable correlation (*r* = 0.71) compared with the correlation with BAI (*r* = 0.63, *z* = 1.80, *p* < 0.05). Notably, these correlations were medium and remained significant after correcting for other DASS‐21 subscales, partial *r* = 0.41, and 0.39 of DASS‐21 depression and anxiety, respectively, indicating the acceptable specificity of these two subscales.^1^ These findings suggest that the DASS‐21 has convergent and divergent validity. Finally, the total DASS‐21 score similarly correlates with BAI and BDI‐II.

### 4.5 Discriminate validity based on the HTMT approach

Henseler et al.^41^ proposed using the HTMT ratio of the correlations as another strategy for determining discriminant validity. The HTMT_0.85_ criterion revealed a discriminate validity problem in the DASS‐21 constructs of depression stress (*r* = 0.93) and anxiety stress (*r* = 0.87), thereby indicating a multicollinearity problem in these latent constructs. This indicated that the majority of the item constructs measure the same content. Only the depression anxiety scale outperforms the HTMT_0.85_ criterion (*r* = 0.81), indicating that depression and anxiety are distinct constructs in DASS‐21.

## 5 DISCUSSION

The study aimed to assess the factor structure and psychometric properties of the Jordanian version of the DASS‐21 scale. In terms of the factor structure of the DASS‐21, different measurement models were compared through CFA. The results showed a significantly better fit for the bifactor model with one general factor and three specific factors (depression, anxiety, and stress), a finding that is consistent with the results of several recent studies.^1^, ^7^, ^10^, ^23^, ^32^, ^34^, ^55^, ^56^, ^57^, ^58^ This finding may resolve the controversy and disparity with respect to the factor structure of the DASS‐21 scale, as most studies conducted in the Arab world, despite their scarcity, produced mixed results; some of them identified one common factor for general distress^40^ or two factors,^37^ whereas others supported the original structure with three correlated factors^39^ and still others highlighted the need for versions of DASS‐21 with 20 items,^36^ 18 items, 17 items,^40^ or 16 items.^37^ The current study, which is the first in Jordan, aligns with the latest results of previous international studies, as it confirms the importance of the bifactor structure without deleting any item from the original scale. This renders it possible to make comparisons with other cultures, thereby making the current study a qualitative addition to the global knowledge repository in this field. Notably, the items that exhibited non‐significant loadings on their factors (5, 9, and 14) were similarly repeated in previous studies.^1^, ^32^ Herein, it should be noted that items (5, 13, 9, 6, 11, and 14) from the Jordanian version of the DASS‐21 did not have significant loadings on their relevant factors. However, these items were preserved and not removed from the scale for the following reasons: first, the bifactor model provided the best internal structure fit to the observed Jordanian data. The loadings of all items on the general factor were significant and large (>0.44). This common component was considered a measure of a global construct trait named “general distress,” which has been repeated in recent investigations across different cultures.^1^, ^23^, ^32^, ^33^, ^34^ Second, removing such items would make it more difficult to perform cultural comparisons, as most internationally validated versions of the DASS‐21 have 21 items.^1^, ^7^, ^14^ Third, the internal consistency of the total score and the subscales was excellent, and removing any item did not seem to improve the scale's reliability.

In terms of psychometric characteristics, the Jordanian version showed optimum reliability for the subscales and excellent reliability for the total score. This finding is consistent with previous investigations.^1^, ^8^, ^9^, ^10^, ^12^, ^14^, ^15^, ^16^, ^27^, ^29^ The inter‐correlations between the three DASS‐21 scales were high and consistent with previous research,^1^, ^14^, ^27^ thereby indicating a large overlap in the content of these scales and the presence of a common general factor. Many studies have found such strong correlations.^9^, ^11^, ^14^, ^59^ As a result, while the DASS‐21 has three distinct factors, it prefers to use the total score rather than the subscale scores.^23^ The hierarchical omega (*ω*_H_) for the three subscales is too small, which is consistent with Chen et al.^16^ finding among Chinese teachers. Given these small values of the ω_H_ coefficients, the presence of the general factor score and the subscale scores interprets the subscales' ability to accurately indicate the unique constructs as very limited. Due to the general factors, there is very little reliable variance.^53^, ^55^ In other words, using the total score on the DASS‐21 to measure general distress is preferable to using the scores on the three subscales. The correlations of the three subscales with measures of similar–different constructs were generally adequate, consistent with previous studies.^1^, ^7^, ^14^ Therefore, the DASS‐21's convergent and divergent validity was supported by the unique correlations between it and other relevant clinical instruments.

Furthermore, the correlation between the anxiety subscale and the BAI was unique, recording the highest correlation value in the correlation matrix. This was the same case for the correlation between the depression subscale and the BDI‐II, indicating that these two subscales measure depression and anxiety. The stress subscale had nearly equal correlations with the BDI‐II and the BAI, indicating that stress is a broad and common concept among depression and anxiety, as its symptoms are linked to both.^5^ Notably, the Jordanian participants reported higher scores of depression, anxiety, and stress levels compared to the respondents in the majority of previous studies, especially on the stress scale, like the studies of Bottesi et al.^1^; Henry and Crawford^7^; and Alfonsson et al.^10^ The Jordanian version of the DASS‐21 revealed a strong correlation between the total scores and the specific external indicators of anxiety and depression. This is in line with the findings of Bottesi et al.^1^ and Osman et al.^23^ and the results of factor analysis, which revealed that the total score could be used to measure general distress in research investigations, where all items in each subscale were low to moderately loaded. However, their loading on the general factor is higher than their loading on their subscales. This result is consistent with previous research findings.^1^

In one of the few studies that evaluated the discriminant validity of DASS‐21 by using the HTMT technique, the results confirmed the discriminant validity of the depression anxiety scale, implying that depression and anxiety, as measured by the DASS‐21, are two distinctive latent traits. This finding is consistent with the current study's divergent validity of correlations with similar–different constructs and the results of previous research.^60^ It is also compatible with the bifactor structure of the DASS‐21. However, it was found that the DASS‐21 encountered problems with discriminant validity in terms of depression stress and anxiety stress, implying that stress symptoms are intrinsic to depression and anxiety. Therefore, the scale could not identify stress as a distinct construct due to the overlap in the contents of these traits. This result is relatively consistent with the findings of the study conducted by Laranjeira et al.^60^ This conclusion was supported by the items loading on the stress subscale in the bifactor solutions. This result is partially consistent with the findings of the study conducted by González‐Rivera et al.^24^ DASS‐21 has substantial psychometric deficiencies, particularly in discriminating validity in Puerto Rico's Hispanic community, and in line with Chen et al.^15^ findings among different Chinese populations. This result was unsurprising given the high correlation between anxiety and stress as well as between the depression and stress constructs.

## 6 LIMITATIONS AND RECOMMENDATIONS

Although the current study assumed the validity and reliability of the Jordanian version as a measurement tool, clinical samples were not included. Subsequent studies must include other psychological tools to confirm the scale's divergent and discriminant validity. It has also been assumed that different types of individuals or participants will be included, emphasizing the importance of increasing the sample size and developing norm scores for Jordanian people to interpret performance on the scale.

## 7 CONCLUSION

The DASS‐21 factor structure was evaluated, and its psychometric properties were tested in the Jordanian cultural context. The bifactor model best fits the Jordanian sample data. The convergent validity and internal consistency of the DASS‐21 were supported. However, it was found that the discriminant validity of DASS‐21 was insufficient. We recommend that, in addition to using the DASS‐21 total score to detect general distress, it must also be permissible to use the depression and anxiety subscale scores. However, a stress subscale must be cautiously used to detect stress symptoms in Jordanian adults. This tool, which has optimum psychometric properties, is anticipated to assist researchers and psychologists in quickly assessing general distress symptoms.

## FUNDING INFORMATION

This research received no particular grants from public, commercial, or non‐profit funding entities.

## CONFLICT OF INTEREST STATEMENT

The authors declare no conflict of interest.

## ETHICS STATEMENT

Approval of the Research Protocol by an Institutional Reviewer Board: N/A.

Informed Consent: No patient contact was involved in this project.

Registry and the Registration No. of the Study/Trial: N/A.

Animal Studies: N/A.

## Data Availability

The data that support the findings of this study are openly available in figshare at https://figshare.com/s/a9d49e5dd2eae13d2b10, doi: 10.6084/m9.figshare.25024415
